# Fighting Dog-Mediated Rabies in Namibia—Implementation of a Rabies Elimination Program in the Northern Communal Areas

**DOI:** 10.3390/tropicalmed5010012

**Published:** 2020-01-17

**Authors:** Rauna Athingo, Tenzin Tenzin, Albertina Shilongo, Emmanuel Hikufe, Kenneth K. Shoombe, Siegfried Khaiseb, Jolandie van der Westhuizen, Moetapele Letshwenyo, Gregorio Torres, Thomas C. Mettenleiter, Conrad M. Freuling, Thomas Müller

**Affiliations:** 1 Animal Disease Control, Subdivision-North West, Directorate of Veterinary Services (DVS), Ministry of Agriculture Water and Forestry, Ongwediva 15006, Namibia; pndinelao@yahoo.com (R.A.); kshoombe@gmail.com (K.K.S.); 2 World Organisation for Animal Health (OIE), Sub-Regional Representation for Southern Africa, Gaborone 25662, Botswana; t.tenzin@oie.int (T.T.); m.letshwenyo@oie.int (M.L.); 3 Directorate of Veterinary Services (DVS), Ministry of Agriculture Water and Forestry, Windhoek 12022, Namibia; Albertina.Shilongo@mawf.gov.na (A.S.); emmanuel.hikufe@mawf.gov.na (E.H.); 4 Central Veterinary Laboratory, Directorate of Veterinary Services (DVS), Ministry of Agriculture Water and Forestry, Windhoek 13187, Namibia; Siegfried.Khaiseb@mawf.gov.na (S.K.); jolandie.pienaar@gmail.com (J.v.d.W.); 5 World Organisation for Animal Health (OIE), Science Department, 75017 Paris, France; g.torres@oie.int; 6 Institute of Molecular Virology and Cell Biology, Friedrich-Loeffler-Institut, 17493 Insel Riems, Greifswald, Germany; thomas.mettenleiter@fli.de (T.C.M.); conrad.freuling@fli.de (C.M.F.)

**Keywords:** rabies, lyssavirus, dog rabies, elimination program, Namibia

## Abstract

The major part of the global burden of dog-mediated rabies falls on Africa and Asia, where still an estimated 60,000 people die of the disease annually. Like in many African countries, dog-mediated rabies is a major public health concern in Namibia, costing the country an estimated 242 human deaths during the past two decades, in particular in the Northern Communal Areas (NCAs). Consequently, under the “One Health” concept, the Namibian government adopted a National Rabies Control Strategy in 2015, which strives to contribute to the global goal of ending dog-mediated human rabies deaths by 2030. A key component of this strategy was the implementation a dog rabies elimination program in the NCAs in 2016, being designed as a stepwise regional rollout strategy by building on experience gained in a pilot project area. The area of implementation covers approximately 263,376 km^2^ and 64 constituencies, with around 1.2 million inhabitants and estimated 93,000 dogs.

## 1. Introduction

Rabies is a devastating viral infection of animals and humans that is caused by members of the Lyssavirus genus in the family Rhabdoviridae [1]. Rabies virus (RABV), the prototype lyssavirus mainly associated with domestic dogs, is responsible for over 99% of all estimated 59,000 (95% CI: 25–159,000) human rabies deaths per annum, with the highest burden of disease in developing, low, and middle income countries of Asia and Africa. With 3.7 million disability-adjusted life years (DALYs) and 8.6 billion United States (US) dollars of economic losses annually, dog-mediated rabies ranks third in the list of burden of neglected tropical diseases [2,3], with the highest per-person death rate occurring in the poorest countries in sub-Saharan Africa [2,4].

Mass vaccination of dogs as a key component of national rabies elimination programs has been successful in eliminating dog-mediated rabies in Europe, North and Latin America, and Japan [5,6,7]. In Africa, dog mass vaccination programs showed in principle some success, i.e., KwaZulu-Natal, South Africa [8], Serengeti, Tanzania [9,10], and Malawi [11]. However, dog rabies control across Africa is largely non-existent, or is hampered by various reasons including lack of resources [12,13]. 

Namibia, which is home of about 2.1 million people, belongs to those African countries for which both dog- as well as wildlife-mediated rabies cycles have been verified [14]. At the turn of the 19th to the 20th century, rabies was virtually unknown in the country [15,16]. The earliest records date from the year 1887 which is referred to by the Herero people as “Otjorundumba”, or “year of rabies”, and during which significant numbers of cattle died presumably from dog-mediated rabies [15,16]. Until the end of the 1940s, rabies was only reported from the Northern communal areas (NCAs). In subsequent years, the disease rapidly spread south, also involving black-backed jackals (*Canis mesomelas*) as a new wildlife reservoir host, eventually causing spill-over infection in domestic and wildlife ruminants [17,18]. Between 2011 and 2017, more than 400 rabies cases were detected in 17 different wildlife species [14]. Since the mid of the 1970s, although rabies was regarded as endemic throughout Namibia [16], dog-mediated rabies was mostly restricted to the NCAs [14]. These areas, which cover approximately 41 per cent of the total area of Namibia, accommodate about 60 per cent of the human population. Despite interventions, e.g., free annual rabies vaccinations in dogs, dog-mediated rabies remained endemic, and the number of reported rabies cases in dogs unchanged. As a result, between 2001 and 2017, a total of 242 human rabies deaths were recorded, with the majority of these victims being children. Poor public awareness, inadequate surveillance and reporting of rabies cases, underestimated dog populations, low vaccination coverage, as well as a lack of stakeholder involvement and research on the disease contributed to this devastating situation [14]. 

These unnecessary human case fatalities prompted the Namibian government to develop a national rabies control strategy towards the elimination of dog-mediated rabies in the country [14], which strives to contribute to the global goal of ending dog-mediated human rabies deaths by 2030 [4]. Here, we address the successes, challenges, and pitfalls experienced, as well as the lessons learned during the implementation of the dog rabies elimination program in the NCAs for scaling up in the near future. 

## 2. Materials and Methods 

### 2.1. Development and Implementation of a Rabies Control Strategy

Following the “One Health” concept, a national rabies control strategy, adopted in March 2015, was collectively developed by the Ministry of Agriculture Water and Forestry (MAWF), the Ministry of Health and Social Services (MoHSS), and the Veterinary Association of Namibia (VAN) based on an assessment while using the Stepwise Approach towards Rabies Elimination (SARE) [19,20]. The strategy aims at providing a framework for effective and sustained rabies control at a national level with the ultimate goal to eliminate dog-mediated rabies in the NCAs [14]. The overall objectives of this strategy encompassed a critical revision of previous rabies control efforts and legal provisions on rabies control, including enforcement procedures as well as guidelines and standard operating procedures. Moreover, the strategy also aimed at raising public awareness, improving rabies surveillance, establishing baseline information on dog population densities and management, providing adequate infrastructure and resources, strengthening intersectoral collaboration and international co-operation in rabies control, and enhancing rabies research at the national level. 

### 2.2. Program Areas and Implementation

**Figure 1:**
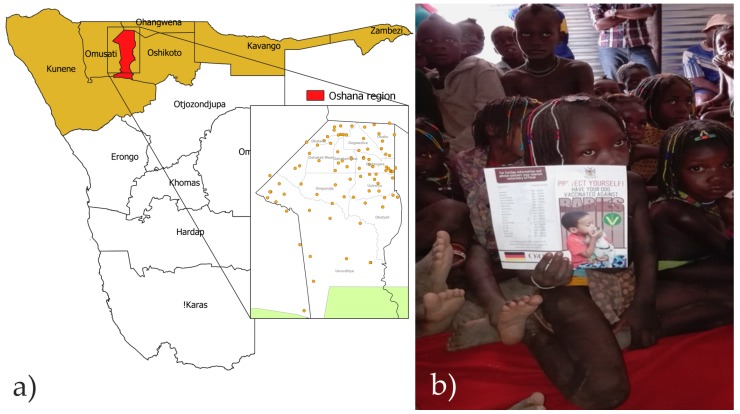
(**a**) Map of Namibia with the administrative boundaries of the regions. Insert: Vaccination points in the Oshana region and distribution of constituencies. (**b**) Photograph during school awareness in the Oshana region with a child holding the information leaflet.

The implementation of the dog rabies elimination program was carried out in two phases: (i) a pilot phase (March 2016–March 2017) and (ii) a rollout phase (April 2017–March 2018). The pilot project was set up in the Oshana region, the smallest of the eight regions in the NCAs comprising 8647 km^2^. The area is a centre of major economic activities comprising the three towns of Ondangwa, Ongwediva and Oshakati, and it has a human population of 175,000 (20 inhabitants/km^2^) living in 12 constituencies (Figure 1). The pilot project was launched on 02 May 2016, with both national and international participation. In the rollout phase launched on 06 April 2017 in the Omusati region, vaccinations expanded to the entire territories of NCAs comprising the regions of Oshikoto, Omusati, Ohangwena, Kunene, Kavango East, Kavango West, and Zambezi, aiming at establishing well managed mass dog vaccination campaigns in each district on an annual basis. This entire area of implementation covered 263,376 km^2^, with approximately 1.2 million people living in 64 constituencies [21] (Figure 1). 

### 2.3. Program Management

The Directorate of Veterinary Services (DVS) of MAWF planned and managed the dog rabies elimination program in collaboration with other stakeholders, including MoHSS, Ministry of Education (MoE), the Central Veterinary Laboratory (CVL), and the University of Namibia (UNAM) School of Veterinary Medicine. Technical assistance was provided through international partners, i.e. OIE headquarters, Paris, France, the OIE Sub-Regional Representation for Southern Africa, Gabarone, Botswana, and the Friedrich-Loeffler-Institut (FLI), Germany. The program received substantial financial support from the German Ministry of Food and Agriculture (BMEL). A national rabies program coordinator was appointed, whose field of responsibility included the arrangement of awareness and education campaigns, the planning and execution of mass dog vaccination campaigns, the recruitment and training of vaccination teams, and the drafting and distribution of timely reports to all stakeholders, other cooperation partners, and the general public, to manage implementation of the program at the regional level. 

Human rabies prevention was coordinated by the Namibian Public Health Institute (NPHI) and managed by public health officers at a regional level.

### 2.4. Awareness and Educational Campaigns

Educational material for awareness campaigns was obtained from the Global Alliance for Rabies Control (GARC), the World Society for the Protection of Animals (WSPA), MAWF, and the OIE. Information was mainly conveyed through public communication while using media channels (television, radio, newspapers), display of posters and banners, distribution of leaflets and other materials to students, teachers, and the general public before any vaccination campaign. Additionally, regional symposia, meetings, and seminars for constituency councils and regional stakeholders, e.g. public health services, administrative and financial staff, paramedics, were organized to enhance the impact of awareness campaigns. Rabies awareness and education was included into the general integrated school health program in close collaboration with the Ministry of Education, Arts, and Culture (MoEAC) and MoHSS. 

### 2.5. Training

Regular training sessions for vaccination teams, district veterinary officers, district medical officers, health workers, and laboratory staff were organized in preparation of, and prior to, vaccination campaigns. The addressed topics included rabies related epidemiological information, state-of-the-art post-exposure prophylaxis, stray dog and refuse management, dog handling and behaviour, vaccination techniques, and the importance of adequate surveillance. As for the latter, special training and material for sample collection were provided to vaccination teams and district veterinary officers to enhance surveillance in the NCAs.

### 2.6. Mass Dog Vaccination Campaigns

High-quality inactivated vaccines compliant with OIE standards (Rabisin^®^, Boehringer-Ingelheim, Germany) were procured through the OIE Vaccine Bank [22,23]. Cold chain of the vaccine was maintained (2–8 °C) during transport and storage according to the manufacturer’s instructions. Vaccination campaigns were announced well in advance by posters and radio broadcasts, and at the day of vaccination attention was captured again while using loud speaker announcements. 

Static point vaccination was the method of choice utilizing the dense network of fixed cattle crush pens (on average 1–3 km apart) in each constituency (Figure 1, insert). In densely populated urban areas, additional door-to-door vaccination of owned dogs as well as catch-vaccinate-release of stray dogs whenever possible was conducted by mobile vaccination teams. The vaccination campaigns were scheduled during school holidays. Vaccination teams worked simultaneously, with each team consisting of 4–5 and 2–3 vaccinators in the pilot and rollout phase, respectively, including a state veterinarian, animal health technicians, and recruited vaccination staff. Apart from concerted mass dog vaccination campaigns, vaccination of dogs was also conducted alongside regular cattle vaccination for livestock diseases, such as contagious bovine pleuropneumonia (CBPP) and foot and mouth disease (FMD). The vaccination of dogs was provided free of charge with owners receiving a vaccination certificate in official language. Previously trained staff members that were involved in mass dog vaccination campaigns received rabies pre-exposure prophylaxis. The collection of campaign related data included records on the number of vaccinated dogs versus number of dogs presented and not presented for vaccination. 

### 2.7. Rabies Surveillance and Diagnosis

In the frame of rabies surveillance, suspected rabid animals were reported to DVS by veterinarians, para-professionals, and farmers [14]. For laboratory confirmation, the samples were submitted to the CVL in Windhoek or the regional branch of CVL in Ondangwa (Government Notice 46 of 2011) and tested while using standard diagnostic test, i.e., FAT, for rabies [24]. The data were compiled in the national database for animal diseases and analyzed by the Division of Epidemiology, Surveillance & Import/Export Control at DVS [14]. Suspect human rabies cases were mainly diagnosed on clinical reasons and reported by public health authorities to MoHSS. 

## 3. Results

### 3.1. Pilot Project Phase

During the pilot project phase (March 2016–March 2017), more than 6000 leaflets and 500 posters were distributed to schools and public places in the Oshana region in the frame of awareness campaigns. Additionally, two television and four radio reports were broadcasted. In total, nine local stakeholder meetings were carried out in Ondangwa, Ongwediva, and Oshakati. As part of the school-based education program, awareness campaigns were held in 119 of 137 schools (86.9%) that were located in the 11 constituencies of the pilot project area, which reached approximately 44,000 students. 

Two mass dog vaccination campaigns were carried out by eight vaccination teams. During 46 vaccination days of the first campaign (02 May–19 June 2016) a total of 24,638 dogs and 2078 cats were vaccinated. While vaccination efficiency per team ranged between 270 and 423 animals per week, one team vaccinated a record number of 584 animals in one week. The number of dogs vaccinated during this first campaign exceeded the projected dog population of 12,000 by a factor of three. A review of dog population estimates after the first campaign revealed at least 27,900 pets to live in the pilot project area, which resulted in an estimated vaccination coverage of 95.7%. In the second campaign comprising 32 vaccination days (24 October–7 December 2016), a total of 12,634 dog vaccinations were recorded, of which 6369 (48.5%) dogs were vaccinated for the first time attaining an estimated 68.3% coverage for the second campaign. In addition, 907 cats were vaccinated during this campaign. During both campaigns, more than 96.0% of the vaccinations were performed at static point vaccinations. 

### 3.2. Rollout Phase

During the rollout phase (April 2017 and March 2018), more than 3500 rabies posters and 35,000 leaflets were distributed throughout the NCAs. School-based awareness campaigns were only conducted in the Omusati and Zambezi region, reaching more than 12,500 students in 42 schools, while television and radio reports and newspaper articles were broadcasted and released in all eight regions, respectively. Stakeholder meetings were conducted in the districts of Ohangwena, Kavango East, and Kavango West. 

**Figure 2:**
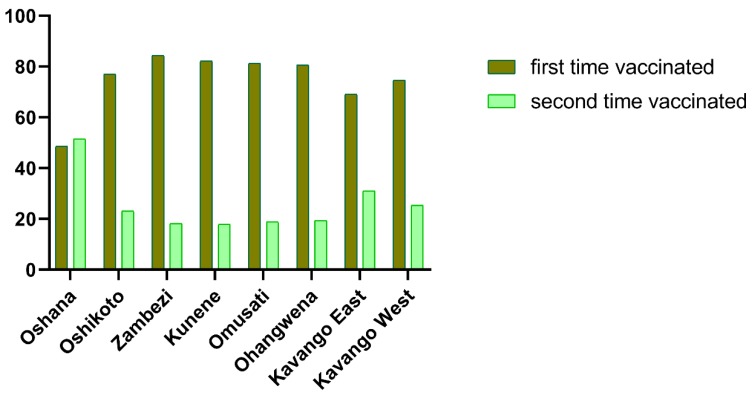
Percentages of dogs presented for vaccination in 2017 from the eight districts of the Northern communal areas (NCAs), stratified according to their vaccination status.

During the years 2017 and 2018, the number of vaccinated dogs in the NCAs amounted to 99,814 and 72,953 dogs, of which 61,195 (61.3%) and 38,350 (52.6%) dogs were vaccinated in the frame of targeted mass dog vaccination campaigns, respectively. The remaining 76,969 dogs were vaccinated alongside regular cattle vaccination for livestock diseases. In 2017, between 48.5% and 84.3% of the dogs received first time vaccination, depending on the region (Figure 2). The vaccination coverage in this year was estimated to range between 26% and 74%, depending on the region, with highest coverage in the Omusati district (Table 1). The minimum vaccination coverage in relation to the estimated pet population (Table 1) would be 39.2%, on average. In addition, 10,538 and 8710 cats were vaccinated in all districts during 2017 and 2018, respectively. 

### 3.3. Rabies Surveillance

**Figure 3:**
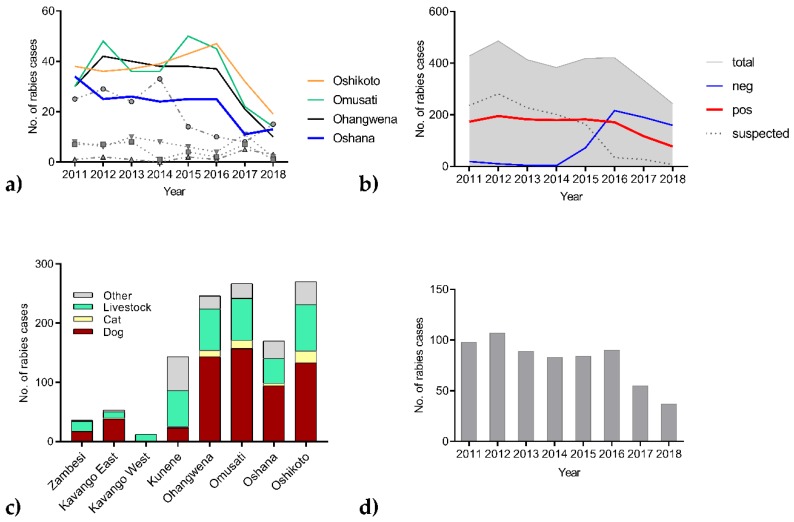
Rabies surveillance data of 24 different animal species from the eight districts of the Northern Communal Areas (NCAs) for the time period 2011–2018, (**a**) number of animals tested positive for rabies differentiated by region. The pilot project area of Oshana and adjacent regions (see Figure 1a) are highlighted, the numbers for the other regions are also provided: Kunene (circle), Kavango East (triangle down), Kavango West (triangle up), Zambesi (square) (**b**) numbers of laboratory confirmed rabies cases, samples tested negative and suspected animals, (**c**) cumulative number of rabies cases per region and species, and (**d**) number of reported rabies cases in dogs.

**Figure 4:**
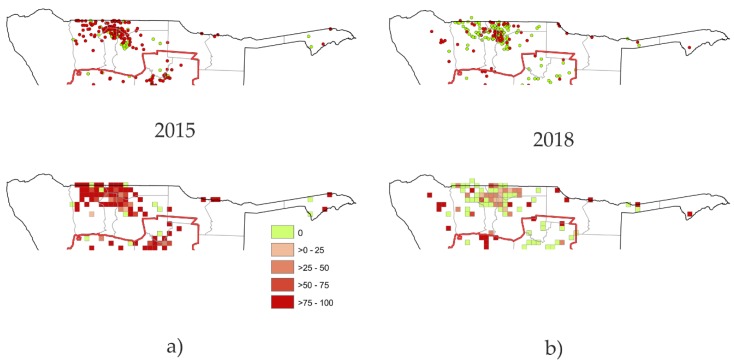
Rabies surveillance data from the eight districts of the Northern Communal Areas (NCAs), (**a**) 2015, and (**b**) 2018. Laboratory confirmed rabies cases are indicated as red dots whereas samples with a negative test results are colored in greed (upper maps). Lower panel: Percentages of rabies positive samples within grid cells of 20 km × 20 km.

The analysis of the animal rabies surveillance data showed a declining trend for the number of reported rabies cases for the central part of the NCAs, whereas a stable trend was observed in the other regions (Figure 3a). While the number of reported suspect animals declined, there is an increase in the number of animals that tested negative for rabies. Rabies cases in dogs were mostly detected in the Oshana, Oshikoto, Omusati, and Ohangewena region (Figure 3c), with a reduction from over 90 cases between 2015 and 2016 to 34 in 2018 (Figure 3d). When the rabies surveillance data were visualized on a map, the majority of cases were located in the central part of the NCAs. While using the percentage of positives per area unit (grid cells with a length of 20 km), the decrease from 2015 to 2018 was demonstrated (Figure 4). Human rabies deaths showed also a declining trend, from 23 cases in 2015, 13 cases in 2016, six cases in 2017, to only one case in 2018. 

## 4. Discussion

This study reports the implementation of mass vaccination of dogs across a large region in Africa, and joins other attempts to control dog mediated rabies in Southern and Eastern Africa [8,11,25,26]. A challenge to the elimination of rabies is the difficulty of developing and implementing vaccination programs that enable large numbers of dogs to be vaccinated within a short time period [11]. There are only a few countries in Southern Africa and Asia, where large numbers of dogs were vaccinated as part of governmentally supported dog rabies elimination programs [11,26,27,28,29,30,31]. 

There are various ways to retrospectively evaluate the success and outcome once such a program is implemented and campaigns are conducted. One possibility would be the sole analysis of the surveillance data, another a list of topics and areas to optimize. Such approaches have their advantages, but fall short in capturing the entire complexity of this mission and considering that implementation of such a huge program is a success in itself. Particularly, the willingness of people to bring dogs to the vaccination point is exemplary, and it was only possible through public awareness prior to the vaccination. Well-coordinated community and public awareness campaigns through various media, such as print media, television, radio, and other electronic communication devices, played a crucial role in sensitizing the community regarding rabies as a zoonotic disease and the importance of vaccination of pets. Including rabies education in the integrated school health program of elementary schools had a tremendous positive impact on the success of vaccination campaigns notably in the pilot project region as children in the NCAs are usually responsible for the caring of dogs in their families. This had not only a short term effect, but likely and hopefully will pave the way for a future setting, where mass vaccinations are no longer needed, as the majority of owned dogs is per se vaccinated as an effect of responsible dog ownership and decreasing poverty [32]. The strong political will to fight dog-mediated rabies in the country was another cornerstone in this program, as reflected by the long-term national rabies control strategy, the human and financial resources provided by the Namibian government, and the resulting interaction with relevant stakeholders identified in the action plan. The project received appreciation from upper level politicians through to all members of communities, which helped to disseminate information and increased the overall motivation. Particularly, the early and constant involvement of traditional leaders was crucial for acceptance and outreach.

This was the first large-scale One Health program in Namibia, encountering infrastructure and human resource challenges, as also experienced before under similar socio-economic settings [26]. These challenges included logistical shortcomings in transportation, i.e. some vaccination points were missed or could not be reached, or shortages of some resources (e.g., identification means for vaccinated dogs, printing facility for vaccination certificates, and other relevant documents) to be used during the campaign. Additionally, in 2017 and 2018, unforeseen budgetary constraints at the governmental level had a negative impact. For instance, late payment of daily allowances, overtime work, and low salaries for recruited vaccination staff negatively affected the morale of staff members. As a result, although vaccination campaigns were scheduled during the general school holidays to ensure high degrees of acceptance among dog owners, there were timely delays in many regions. Furthermore, there were sometimes no teams available to conduct awareness campaigns during the roll out phase. This affected school-based awareness campaigns in five regions, as well as general publicity of the rabies campaigns. In general, it was decided to focus all efforts on actually vaccinating as many dogs and cats as possible and omit further assessments on the actual vaccination coverage due to these constraints. Obviously, this approach hampers any study to quantify the effectiveness of vaccination intervention, but appears to be a common limitation since in some published studies on mass dog vaccinations post vaccination surveys are not included [13]. The only measure would be the direct impact of the vaccination on disease transmission via the number of detected rabies cases. However, rabies surveillance is heavily influenced by the awareness and vigilance and, in fact, education on rabies was a cornerstone of the rabies pilot project. Based on surveillance records, it is evident that both the numbers of suspect animals and confirmed rabies cases decreased after the implementation of the vaccination (Figure 3a,d). While we cannot exclude that this is fluctuation independent of vaccination, the increasing number of animals that tested negative (Figure 3b and Figure 4) is a very positive indicator for improved surveillance and also provides an indication that the Namibian rabies control efforts already have a positive effect in eliminating dog-mediated rabies from the NCAs. 

For the purpose of planning, e.g. vaccine procurement, budgeting, etc., the proper estimation of the dog population is a prerequisite [33]. A survey that was conducted in parallel to vaccination revealed that the number of dogs counted and vaccinated in the Oshana region during the pilot phase (Figure 1) exceeded the projected 12,000 by a factor of three, clearly indicating that the dog population of the region had been greatly underestimated over the years. Similarly, the projected pet population for the entire eight regions of NCAs during the roll out phase of the project also exceeded previous estimates to a total of about 159,000 pets, of which 90% are assumed to be dogs (Table 1). Based on a reassessment of this data, the overall estimate for the human-dog ratio for the NCAs is 8.3, while the ratios may vary from 7.0 to 11.6, depending on the region. This is in general agreement with the estimates for the South African Development Community (SADC) region [34]. However, the observation that mainly young dogs were taken to vaccination and a relatively high percentage of dogs received first time vaccination (Figure 2) points to a high dog population turnover, so that, even the 2016 pet population data may be an underestimation of the real situation. This is supported by data from the Omusati region where more dogs were vaccinated in 2017 than established by the 2016 survey of the population (Table 1). If these numbers are taken into consideration, then the human-dog ratios are closer to estimates from rural areas in similar settings, like Zimbabwe (4.5–4.7, [35,36]), Zambia (6.7, [37]), or Tanzania (6.3, [9]). 

While in this project, the higher demands for vaccines were buffered by rather flexible procurement procedures through the OIE vaccine bank [22,23], other rabies vaccination projects, where the dog population was severely overestimated faced problems. For instance, in Tanzania the procurement of larger than needed quantities of dog vaccines resulted in a storage for long periods of time, thus blocking a large proportion of the total budget and limiting the cost-effectiveness of the program [26]. Dog ecology studies should ideally be conducted in the early stages of project planning, as both dog and human populations can be very dynamic and may change over time [38]. In any case, there is a need for revision to establish reliable local dog population estimates for the NCAs for future campaigns while using established methodology [11,39].

In Namibia, more than 95% of the dogs are owned and would be accessible for parenteral vaccination based on the socio-cultural background of the ethnic groups living in the NCA’s, while, in contrast, the number of stray dogs is extremely low. Therefore, similar to other African campaigns, Namibia predominantly applied static point vaccination as the method of choice for dog vaccination. However, the ability of this approach to reach a sufficient proportion of dogs in all community settings is controversially debated [9,34,40,41]. In fact, the proportion of dogs being presented by the public is very volatile and might depend on social, cultural, geographic, and economic factors [41]. 

One parameter is the human population density, i.e. the number of people per km^2^, which is extremely low in most parts of the NCAs when compared to other African regions (Table 1) with similar large-scale vaccination programs, like in Uganda (145 people/km^2^, [28]), KwaZulu Natal (108 people/km^2^, [25]), and Tanzania (45 people/km^2^, [26]). Additionally, with 263,376 km^2^ the area of implementation in Namibia is by far larger than in any of the other regions. Hence, establishing static point vaccination in the NCAs is a great logistical challenge per se, as longer distances have to be covered and more vaccination days are needed, especially if only limited resources are available. Unlike with annual cattle vaccinations for which crush pens at a distance of 5–10 km apart were working well, it soon became evident that the owners were discouraged to travel long distances through the bush land to present their dogs for rabies vaccination. Consequently, in some regions, additional static vaccination points (2–3 km apart) in combination with door-to-door vaccination had to be set up during the rollout phase. Additionally, one vaccination team had to cover several static vaccination points a day because of the relatively low numbers of dogs being presented at each vaccination points, resulting in, sometimes, long waiting periods for dog owners that could be stressful when temperatures were high. However, despite these logistic challenges, the approach reported here demonstrated the feasibility of a vaccination of an average of 580 dogs per day during the pilot phase. This number appears low compared to 1760 dogs reached in Malawi [11], or even 4500 dogs vaccinated per day in India [42], but equals the vaccinator efficiency of Uganda with 596 dogs per day vaccinated [28]. 

Both the vaccination coverages at levels (Table 1) that are likely to interrupt the transmission cycle (Hampson et al., 2009) and the resulting decline in the proportion of rabies positives (Figure 3) indicate that Namibia is on its way to control successfully dog-mediated rabies. While we share similar experiences in the implementation of mass dog vaccination campaigns as Uganda [28], there is still room for improvement. For instance, although various data collection tools were available [43,44,45] they were not considered due to the lack of in-country technical expertise and time constraints with regards to implementing the first phase of the project. However, unfortunately, paper recording of dog vaccinations turned out to be even more time and labor consuming and represented a major hurdle for subsequent reporting and adequate epidemiological analysis, in particular in 2018. As a consequence, DVS decided to introduce a GPS supported data capturing tool (Data Logger) and utilize web-based data surveillance as well as the analysis platform that was developed by GARC in 2016 [45,46] to improve and monitor vaccination campaigns in the future.

## 5. Conclusions

Namibia set the ambitious goal to eliminate dog-mediated rabies in the NCAs in the near future. The implementation of such a large-scale elimination program in an extremely sparsely populated area, such as the NCAs, is of an unprecedented nature and represents an exceptional financial and logistical burden for the Namibian society not commensurate with the size and economic strength of the country. Namibia’s experience of implementing a dog rabies elimination program was also showcased and appreciated during an OIE sub-regional seminar on rabies for SADC that was conducted in Windhoek from 10–12 April 2018. To tackle the cross-border rabies problem in the region of the NCAs, which shares a long open border with Angola the Namibian authorities envisage establishing a long lasting cooperation with the Angolan counterparts to improve the understanding of the epidemiology of the disease and encouraging joint rabies control campaigns on both sides of the border. An inter-regional coordination meeting with Angolan participation was held in 2019, and it is envisaged to be carried out on a regular basis by also including Botswanan authorities in the near future. Although lessons learned during the past few years are only too well known and should be applied to any roll out of rabies elimination programs elsewhere in Africa [26], Namibia, like any other country, will learn by experience. Several insufficiencies had been identified that have to be corrected as soon as possible. These include the need to change from a single responsible person for dog rabies control to a dedicated management team with focused responsibilities to deal with the future planning, organization, as well as engagement of other key sectors for the evaluation of rabies vaccination campaigns. The international support was considered to be very helpful and essential, and it is envisaged to be further developed into a long-lasting strategic partnership. As a further step towards this direction, in 2019, a three-year OIE laboratory twinning project between the CVL and the FLI was launched.

## Figures and Tables

**Table 1:** Data on human census, number of dogs and cats vaccinated and estimates for the pet population in the respective regions to derive the vaccination coverage.

Region	Human Census 2011	Area (km^2^)	Population Density (Persons/km^2^)	Estimated Pet Population 2016 #	Human:Pet Ratio #	Estimated Human:Dog Ratio	Dogs Vaccinated 2017	Cats Vaccinated2017	Vaccination Coverage ∞
Kavango	222,500	48,742	4.56	29,600	7.5	8.4	10,175	1310	39%
Kunene	54,300	115,260	0.47	7100	7.6	8.5	4520	264	67%
Ohangwena	245,100	10,706	22.89	27,800	8.8	9.8	17,349	1724	69%
Omusati	242,900	26,551	9.15	34,700	7.0	7.8	33,549	2416	104%
Oshana	174,900	8647	20.23	27,900	6.3	7.0	12,429	798	47%
Oshikoto	161,900	38,685	4.19	23,100	7.0	7.8	14,902	3468	80%
Zambezi	90,100	14,785	6.09	8600	10.5	11.6	6890	558	87%
total	1,191,700	263,376	4.52	158,800	7.5	8.3	99,814	10,538	69%

**^#^** both data sets include dogs and cats ∞ dog vaccination coverage could not be calculated because pet census data could not be segregated into species.
